# Limited effects of preterm birth and the first enteral nutrition on cerebellum morphology and gene expression in piglets

**DOI:** 10.14814/phy2.12871

**Published:** 2016-07-26

**Authors:** Anders Bergström, Sanne S. Kaalund, Kerstin Skovgaard, Anders D. Andersen, Bente Pakkenberg, Ann Rosenørn, Ruurd M. van Elburg, Thomas Thymann, Gorm O. Greisen, Per T. Sangild

**Affiliations:** ^1^ Comparative Pediatrics and NutritionDepartment of Clinical Veterinary and Animal ScienceFaculty of Health and Medical SciencesUniversity of CopenhagenFrederiksbergDenmark; ^2^ Research Laboratory for Stereology and NeuroscienceBispebjerg‐Frederiksberg HospitalsCopenhagenDenmark; ^3^ Veterinary InstituteTechnical University of DenmarkFrederiksbergDenmark; ^4^ Danone Nutricia Early Life NutritionNutricia ResearchUtrechtthe Netherlands; ^5^ Emma Children's HospitalAcademic Medical CenterAmsterdamthe Netherlands; ^6^ Department of Paediatrics and Adolescent MedicineRigshospitaletCopenhagenDenmark

**Keywords:** Enteral and parenteral nutrition, neonatal brain development, postconceptional age, prematurity

## Abstract

Preterm pigs show many signs of immaturity that are characteristic of preterm infants. In preterm infants, the cerebellum grows particularly rapid and hypoplasia and cellular lesions are associated with motor dysfunction and cognitive deficits. We hypothesized that functional brain delays observed in preterm pigs would be paralleled by both structural and molecular differences in the cerebellum relative to term born piglets. Cerebella were collected from term (*n* = 56) and preterm (90% gestation, *n* = 112) pigs at 0, 5, and 26 days after birth for stereological volume estimations, large‐scale qPCR gene expression analyses (selected neurodevelopmental genes) and western blot protein expression analysis (Sonic Hedgehog pathway). Memory and learning was tested using a T‐maze, documenting that preterm pigs showed delayed learning. Preterm pigs also showed reduced volume of both white and gray matter at all three ages but the proportion of white matter increased postnatally, relative to term pigs. Early initiation of enteral nutrition had limited structural or molecular effects. The Sonic Hedgehog pathway was unaffected by preterm birth. Few differences in expression of the selected genes were found, except consistently higher mRNA levels of Midkine, p75, and Neurotrophic factor 3 in the preterm cerebellum postnatally, probably reflecting an adaptive response to preterm birth. Pig cerebellar development appears more affected by postconceptional age than by environmental factors at birth or postnatally. Compensatory mechanisms following preterm birth may include faster white matter growth and increased expression of selected genes for neurotrophic factors and regulation of angiogenesis. While the pig cerebellum is immature in 90% gestation preterm pigs, it appears relatively mature and resilient toward environmental factors.

## Introduction

Preterm birth (<37 weeks gestation) affects around 15 million infants each year and these individuals have an increased risk of developing psychomotor and cognitive defects, especially when born before 32nd week of gestation (Colvin et al. 2004; Blencowe et al. 2013). After preterm birth, white matter injury, caused by inflammation or hemorrhage, is the most common brain pathology (Volpe 2009a,b), yet the pathophysiology and postnatal adaptation of premature brain development is complex and not understood (Krigger 2006). The cerebellum plays a central role for coordination of motor, vestibular, cognitive, and emotional functions (Villanueva 2012) and undergoes significant growth and differentiation during the late fetal and early postnatal period in infants (Dobbing and Sands 1979). It is highly sensitive to environmental factors and postnatal growth restriction after preterm birth (de Kieviet et al. 2012; Kiessling et al. 2013) and accordingly, cerebellum pathology is associated with cognitive and behavioral sequeale, as well as mild motor deficits (Patra et al. 2006; Limperopoulos et al. 2007).

To study the brain responses to preterm birth, it is relevant to have an animal model that allows study of brain tissues after preterm birth, in parallel with the immaturities in other organ systems (e.g., impaired lung, liver, gastrointestinal, cardiovascular, and kidney functions). In contrast to many other species, the pig shows a pre‐ and postnatal growth spurt for the brain, particularly for the cerebellum, that is comparable in timing with that in humans (Dobbing and Sands 1979; Conrad et al. 2012). Preterm pigs, born at 90% gestation, are at high risk of complications arising from their immature gastrointestinal tract and lungs (Sangild et al. 2013; Caminita et al. 2015), but it is not known if complications are also relevant for the developing brain. This question is important for the potential to use the preterm pig as an animal model in neonatal neuroscience. Functional brain deficits in preterm pigs are indicated by their impaired neonatal arousal, physical activity, balance, exploration, and cognitive function, relative to term pigs (Cao et al. 2015; Andersen et al. 2016). These effects may result from the combined effects of shorter postconceptional age and the postnatal consequences of preterm birth, for example, mild hypoxia, metabolic disturbance, and impaired growth. Interestingly, compromised neurodevelopment and cerebellar growth in preterm pigs are supported by full enteral feeding, relative to total parenteral feeding (Choudhri et al. 2014). Normally, preterm infants are gradually transitioned from parenteral to enteral feeding over the first week(s) after birth, but feeding regimens and diets vary widely.

We hypothesized that functional brain deficits in preterm pigs would be paralleled by developmental delays in cerebellar structure and adaptation of cerebellar gene expression, relative to pigs born at full term. Following an initial study to verify relevant functional delays of cerebellar relevance, stereology, large‐scale quantitative PCR analyses, and western blots were used to assess if postnatal development of the cerebellum differed between preterm and term pigs, and whether early initiation of enteral nutrition affected cerebellum maturation.

## Materials and Methods

### Animals and their treatment

All experimental procedures were approved by the National Ethics Committee on Animal Experimentation (protocol no. 2012‐15‐2934‐00193).

#### Experiment 1

To investigate cognition‐related brain functions, preterm pigs from three litters (cesarean section at 106 days gestation, *n* = 17) were compared with pigs born naturally at full term (117–118 days, *n* = 6). The preterm pigs were reared and nourished according to a standard protocol including parenteral nutrition for the first 3 days of life (96–144 mL/kg/h, as described for Experiment 2) combined with enteral feeding with a cow′s milk based formula (32–224 mL/kg/day) until day 23. All pigs were housed individually. Preterm pigs were initially reared in oxygenated and heated incubators before transition to larger cages with a local heat lamp (3). The pigs born at full term were transported to the experimental facilities at 7 days of age and reared and fed in the same way as preterm pigs. Beginning on day 15, both preterm and term pigs were tested daily in a spatial T‐maze (build as a ‘plus maze’ were one arm is sealed off to form a T), previously validated for use in similar‐aged term piglets (Elmore et al. 2012). In the test, pigs had to learn to navigate via extra maze visual cues to obtain an accessible milk reward in one of two reward arms. For each pig, an accessible reward was placed in a fixed maze arm (e.g., east) while an equal amount of inaccessible milk was placed in the opposite arm (west) to mask olfactory cues. All piglets were tested for 6 days (10 trials/session) and the starting position in each trial within a single session was altered (north or south arm) by block randomization which ensured that the starting position was balanced within a single session. By alternating the starting position the pigs were forced to solve the maze by applying an allocentric learning strategy and use the visual cues to reach the learning criterion (80% correct). Pigs from Experiment 1 were not used for any structural or gene expression brain analyses.

#### Experiment 2

One hundred and sixty‐eight pigs (Danish Landrace × Large White × Duroc) from eight sows were delivered by elective cesarean section at 90% gestation (*n* = 112, 106 days gestation) or 100% gestation (*n* = 56, 118 days gestation), as described in detail previously (Sangild et al. 2002). They were immediately transferred for individual rearing in heated and oxygenated incubators to stabilize respiration and body temperature. While still anesthetized from the cesarean section, the piglets were fitted with a vascular catheter (infant feeding tube 4F, Portex, Kent, UK) inserted into the transected umbilical artery and an orogastric feeding tube (6F, Portex). Both were secured to the skin with sutures. The piglets were initially stratified according to birth weight and sex and then randomly allocated within each stratum to receive either total parenteral nutrition (TPN group) or parenteral nutrition supplied with enteral milk nutrition (ENT group). The milk consisted of bovine colostrum (kindly donated by Biofiber Damino, Vejen, Denmark) administered every 3 h. For the TPN group, parenteral nutrition (a modified solution of Kabiven, Vitalipid, Soluvit and Vamin, all kindly donated by Fresenius Kabi, Bad Homburg, Germany) was given at 96 mL/kg/day on day 1, gradually increasing to 144 mL/kg/day on day 5. For the ENT group, enteral nutrition started at 16 mL/kg/day on day 1, increasing to 64 mL/kg/day on day 5, and this was accompanied by a reduction in parenteral nutrition such that the two dietary regimens both provided similar fluid volumes and were iso‐energetic (increasing from 74 to 110 kcal/kg/day over the first 5 days). On day five, following the critical neonatal period, a subset of the pigs was killed and tissues were collected. For the remaining pigs, the parenteral nutrition was discontinued and all piglets were given increasing amounts of full enteral nutrition with raw bovine milk (64–150 mL/kg/day providing 37–70 kcal/kg/day) for 4 days, and then transferred to fortified whole milk powder (150–200 mL/kg/day, Arla Foods, Viby J, Denmark) until day 26. Further details of the rearing procedures are available in a previous publication which also provides more details of the behavioral differences between preterm and term pigs (Andersen et al. 2016).

### Tissue collection

**Figure 1:**
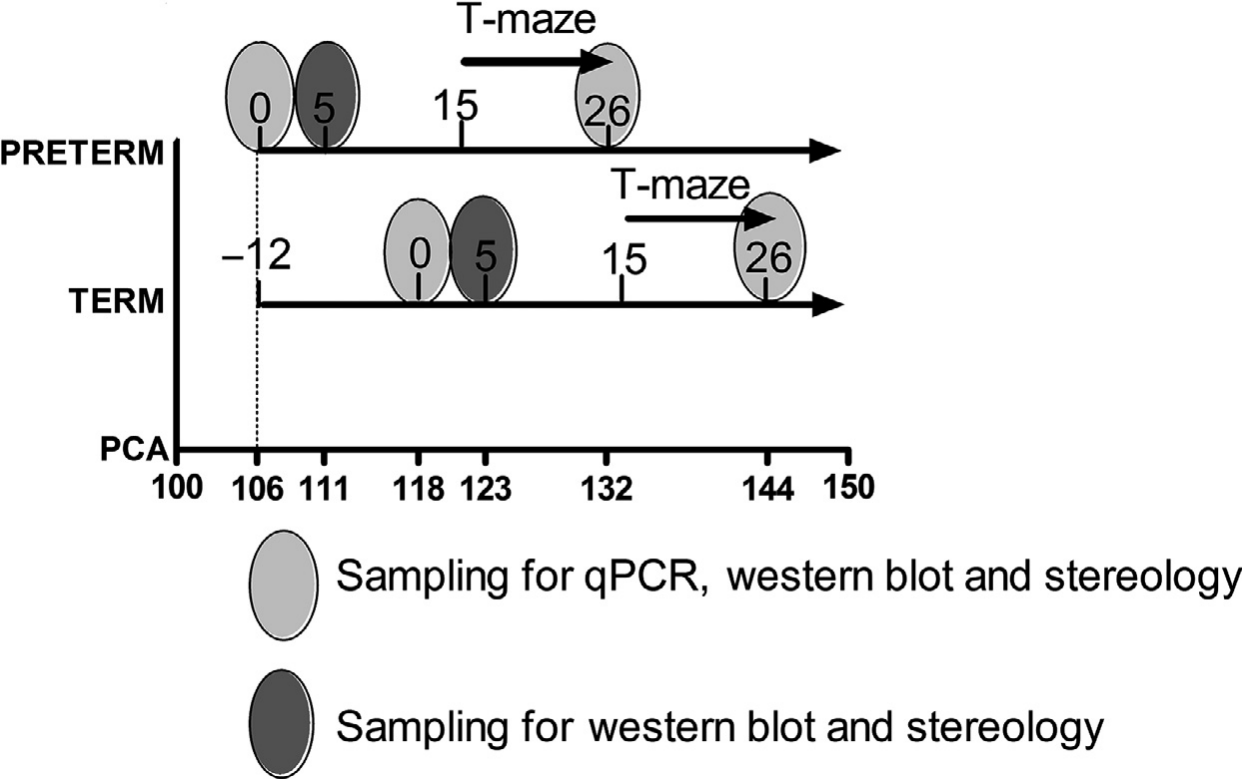
Time lines for Experiments 1 and 2. The numbers on the preterm and term lines indicate postnatal days, whereas the numbers on the postconceptional age (PCA) line indicate postconceptional age, starting at day 106. The 12‐day difference in birth age between the groups is shown as “−12” on the line for term pigs. The T‐maze test was performed only for pigs in Experiment 1. Tissue sampling was done only for pigs from Experiment 2.

Preterm and term pigs were killed at three different time points, day of birth (day 0), day 5 or day 26, and the brains were immediately dissected (Fig. 1). The animals were anesthetized with zolazepam/tiletamin (Zoletil 50, Virbac, Kolding, Denmark), xylazine (Narcoxyl 20 mg/mL, MSD Animal Health, Ballerup Denmark), ketamine (Ketaminol 100 mg/mL, MSD Animal Health), and butorphanol (Torbugesic 10 mg/mL, ScanVet, Fredensborg, Denmark). The anesthetics were mixed and given as a single intra muscular injection at 0.1 mL/kg. When full anesthesia was achieved, the animals were killed with an intracardiac injection of sodium pentobarbital. The brain was carefully divided into two halves by a sharp incision through corpus callosum. All brains were macroscopically evaluated for white matter injury in the periventricular white matter. The right brain hemisphere with brain stem and cerebellum attached were collected intact and immersion‐fixed in 4% formalin for stereological analysis. The left brain part was dissected for snap freezing, including standardized sampling of the cerebellum. These cerebellar samples were immediately frozen in liquid nitrogen and stored at −80°C for subsequent analysis by western blotting and quantitative polymerase chain reaction (qPCR) analyses.

### Stereology on fixed tissue

The stereological evaluation was based on a total of 116 cerebella from the right hemisphere. At day 0, we included *n* = 22 preterm and *n* = 11 term newborn pigs. At day 5, we included *n* = 22 preterm (12 ENT, 10 TPN) and *n* = 22 term (10 ENT, 12 TPN) pigs. At day 26, we included *n* = 17 preterm (8 ENT, 9 TPN) and *n* = 22 term (12 ENT, 10 TPN) pigs. After fixation in 4% paraformaldehyde, the brains were embedded in agar (4% agar in 1.1 mol/L phosphate buffer pH 7.4), and sectioned coronally into 2.1 mm sections. The anterior surface of each section was photographed (EOS 400D DIGITAL, Canon, Søborg, Denmark) and a total of 10–12 sections were obtained per specimen. The volumes were estimated using the Cavalieri's principle (Gundersen and Jensen 1987; Gundersen et al. 1999). Volume measurements were based on the NewCast Stereology software package (Visiopharm, Hørsholm, Denmark) where a point grid is placed randomly on the surface of the sections and the number of points (P) hitting the region of interest (ROI) is recorded. The volume was estimated by multiplying the total number of points hitting the ROI by the area per point, a(p), and by the thickness, T, of the slab, for example, *V* = ∑P *a(p)*T.

### Protein extraction of frozen tissue for Western blots

Western blot analyses were performed on a total of 121 cerebelli from the left hemisphere. From day 0, we included *n* = 10 preterm and *n* = 11 term pigs. From day 5, *n* = 22 preterm (12 ENT, 10 TPN) and *n* = 22 term (10 ENT, 12 TPN) were included. Finally from day 26, *n* = 34 preterm (18 ENT, 16 TPN) and *n* = 22 term (12 ENT, 10 TPN) were included for extraction. For consistency in sampling, the same cerebellar sample was isolated and weighed (<100 mg) and mixed with 1 ml lysis buffer (150 mmol/L NaCl, 1% Triton X‐100, 50 mmol/L Trizma Base, Sigma‐Aldrich, pH = 8.0) and 10 *μ*L protease inhibitor P8340 (Sigma‐Aldrich, Brøndby, Denmark). The tissue was homogenized using gentleMacs dissociator (Miltenyi Biotec, Lund, Sweden). The homogenate was centrifuged for 10 min at 2500 × *g* at 4°C and the supernatant transferred to an Eppendorf tube, followed by another centrifugation. Finally, the supernatant was transferred to cryo tubes and stored at −80°C.

For Western blots, 25 *μ*g protein was run by electrophoresis (Jiang et al. 2008) and primary antibodies against Sonic Hedgehog (Shh), Patched receptor (Ptc), Smoothened (Smo), and Gli Family Zink Finger One (Gli‐1) (Santa Cruz, Heidelberg, Germany) were used. These antibodies detected the protein density for Shh (sc‐9024), Ptc (sc‐9016), Smo (sc‐13943), and Gli‐1 (sc‐20687), respectively. The protein bands were visualized, and the density of the protein bands was detected by Quantity One (Bio‐Rad laboratories, Copenhagen, Denmark).

### RNA extraction for RT‐PCR analyses

RNA was extracted from a total of 77 cerebelli. From day 0 we included *n* = 10 preterm and *n* = 11 term piglets. No RNA was extracted from Day 5 samples. From day 26, *n* = 34 preterm (18 ENT, 16 TPN) and *n* = 22 term (12 ENT, 10 TPN) animals were selected for analyses. Frozen RNA was extracted using the RNeasy Lipid Tissue Mini Kit from Qiagen (Copenhagen, Denmark). Briefly, for each sample a small piece (50–100 mg) of cerebellar brain tissue was dissected on ice and immediately transferred to gentleMacs M tubes (Miltenyi Biotec Norden, Lund, Sweden), containing 1 mL QIAzol lysis reagent. Tissue was homogenized on GentleMacs. 200 *μ*L chloroform was added and tubes were shaken vigorously for 15 sec and transferred to 2 mL Eppendorf tubes followed by centrifugation for 15 min at 12000 × *g*. The upper aqueous phase was transferred to a new Eppendorf tube with 1 volume 70% ethanol and vortexed before transfer to an RNeasy Mini spin column. The RNeasy Lipid Tissue Mini Kit protocol was subsequently followed without optional DNase step and finally RNA was eluted in 40 *μ*L RNase free water. Samples were stored at −80°C until cDNA synthesis.

Total RNA concentration and purity of samples was measured using the NanoDrop ND‐1000 spectrophotometer (Saveen and Werner AB, Sweden) and RNA integrity was assessed using the Agilent Bioanalyzer 2100 and RNA 6000 Nano Kit (Agilent Technologies, Glostrup, Denmark). All RNA integrity values (RIN) were between 5.9 and 8.5. cDNA (Qiagen array, see below) was synthesized using the RT^2^ First Strand Kit provided by Qiagen, using the Stratagene MX3000p according to the manufacturer's instructions. Briefly, the volume of RNA was adjusted to 500 ng and mixed with 2 *μ*L buffer GE and RNase‐free water to 10 *μ*L. This genomic DNA elimination mix was heated for 5 min at 42°C and immediately placed on ice for 2 min. Nine *μ*L reverse transcriptase mix (mixed according to manufacturer's instructions) was then added to the RNA mix and incubated at 42°C for 15 min, followed by 5 min at 95°C. Finally, 91 *μ*L RNase‐free water was mixed with each cDNA reaction. cDNA for Fluidigm qPCR (see below) analysis was prepared by reverse transcription of 500 ng duplicate samples of extracted total RNA using the QuantiTECT Reverse Transcription kit (Qiagen) as described previously (Skovgaard et al. 2013). Nonreverse transcriptase controls were included. cDNA was diluted 1:8 in low EDTA TE‐buffer (VWR, Bie & Berntsen, Denmark) prior to preamplification. Briefly, 5 *μ*L of TaqMan PreAmp Master Mix (Applied Biosystems, Nærum, Denmark), 2.5 *μ*L of primer mix (a 200 nmol/L pool of all primers used in the present study) and 2.5 *μ*L diluted cDNA was mixed and incubated at 95°C for 10 min followed by 15 cycles at 95°C for 15 sec and 60°C for 4 min. Preamplified cDNA was treated with Exonuclease I (16U, *E. coli*) (New England Biolabs, Hitchin Herts, UK) for 30 min at 37°C followed by 15 min at 80°C.

### Quantitative PCR array

Quantitative PCR on Qiagen pathway array was performed on 16 (8 term and 8 preterm) included day 26 samples, all from the pigs that received TPN during the first 5 days. A qPCR master mix was created by mixing 102 *μ*L cDNA, 1248 *μ*L RNase‐free water and 1350 *μ*L 2xRT^2^ SYBR Green Master mix from Qiagen. A volume of 25 *μ*L of the qPCR master mix was then added to all 96 wells of the Human Neurogenesis RT² Profiler PCR Array (Qiagen Nordic, Helsinki, Finland). Array plates were sealed with optical thin wall 8‐cap strips provided by Qiagen. Plates were briefly (2 min) centrifuged at 1000 × *g* to collect contents. All 16 arrays were run with the same amplification program on the Stratagene MX3000p, according to manufacturer's instructions: (1) 95°,10 min (hot start), (2) 40 cycles (95°C, 15 sec, and 60°C, 1 min), (3) 95°C, 1 min and 55°C, 30 sec increasing to 95°, 30 sec (dissociation curve analysis). Results were evaluated by visual inspection of amplification and dissociation curves. Subsequently data (*C*_q_ values) were uploaded to the online PCR analysis tool provided by Qiagen and Sabiosciences (http://pcrdataanalysis.sabiosciences.com/pcr/arrayanalysis.php). Based on this, data were quality controlled. A volcano plot was created by plotting (log_10_) *P* values of a Student's *t*‐test versus (log_2_) fold changes (preterm/term) for each gene. The *P* values were based on Qiagen's array software, which does not correct for multiple testing. Genes fulfilling two criteria: (A) *P* < 0.05 and (B) 40% upregulation (fold change >1.40) or 40% downregulation (fold change <0.714) in the preterm group were selected for further analysis and validation by RT‐qPCR.

### Fluidigm qPCR analyses

Preamplified and exonuclease‐treated cDNA was diluted 1:10 in low EDTA TE‐buffer (VWR, Bie & Berntsen, Copenhagen, Denmark) before qPCR. Expression analysis was performed in two 96/96 Dynamic Array Integrated Fluidic Circuits (Fluidigm, South San Francisco, CA) using TaqMan Gene Expression Master Mix (Life Technologies, Carlsbad, CA), EvaGreen 20X (VWR), and gene‐specific primers as described previously (Skovgaard et al. 2010). Primers were designed in the primer3 software (http://bioinfo.ut.ee/primer3-0.4.0/) using similar criteria as described before (Skovgaard et al. 2010). The following cycle parameters were used for qPCR: 2 min at 50°C, 10 min at 95°C, followed by 35 cycles with denaturing for 15 sec at 95°C and annealing/elongation for 1 min at 60°C. Dissociation curves were generated after each run to confirm the presence of a single PCR product (from 60 to 95°C, increasing 1°C per 3 sec). Nontemplate controls and three interplate calibrators were included on each chip. Reactions were performed in duplicates (cDNA replicates). On each chip, no reverse transcriptase (minus RT) and nontemplate controls (NTC) were included to help trace possible contamination.

A detailed list of genes and associated primer sequences is presented in Table 1. The gene list included 10 genes selected from the Qiagen Neurogenesis array screening (Nrp1, Vegf‐A, Shh, Efnb1, Mdk, Hdac4, Erbb2, Adora2a, LOC100623510, Neurog1). Furthermore, we included additional genes for neurogenesis and angiogenesis (VEGF‐a, VEGF‐b, Dcx, Pdgfr‐beta, Flt1, Flk1, Nrp2, Pxn, Pecam1, Gpr124, Wnt7a, Wnt7b) (Solowska et al. 2002; Krum et al. 2008; Takacs et al. 2008; Kim et al. 2010; Sentilhes et al. 2010; Hatten and Roussel 2011; Hou et al. 2011), cerebellar maturation (Calb1, Itpr3, Gfap, Atoh1, Snf2 h, Syp) (Allais et al. 2007; Flora et al. 2009; Haldipur et al. 2011; Kuypers et al. 2013; Alvarez‐Saavedra et al. 2014), neurotrophic factors (Bdnf, Ntf3, Ngf, TrkB, p75) (Carter et al. 2002, 2003; Johnson et al. 2007), Sonic Hedgehog pathway (Shh, Ptch, Smo1), apoptosis (Lifeguard, Casp3) (Noguchi et al. 2008; Hurtado De Mendoza et al. 2011), hypoxia (HIF‐1a) (Chiral et al. 2004), tight junction integrity (ZO‐1, VE‐Cad, Ocln, Cldn3, Cldn5) (Silwedel and Forster 2006; Sadowska et al. 2010; Luissint et al. 2012; Ben‐Zvi et al. 2014), energy and water metabolism and transporters (Mct1, Mct2, Glut1, Glut3, Aqp4) (Nico et al. 2002; Dienel 2014), and myelination (Mbp) (Ghoumari et al. 2003). With few exceptions, for which validated primers already existed, two primer sets were designed for each gene targeting different locations on the mRNA transcript. We failed to obtain adequate qPCR results for Shh, Syp, Glut1, Nrp3, Hdac‐4, Neurog1, Ngf or LOC100623510.

**Table 1:** Fluidigm qPCR primer list

Gene Symbol/primer	Gene description	Relevance	Sequence 5′ to 3′
Adora2a (P1), F	Adenosine A2a receptor	Qiagen array suggestion	AGCTCCATCTTCAGCCTCCT
Adora2a (P1), R			CCAGTCACCAAGCCATTGTA
Adora2a (P2), F	Adenosine A2a receptor	Qiagen array suggestion	TGCTGAGTGAAGGGAGTGTG
Adora2a (P2), R			TTGAGGCCAGGGGACTCT
Atoh1 (P1), F	Atonal homolog 1	Sonic Hedgehog Pathway	GCCAGTGCAGGAGGAAAGTA
Atoh1 (P1), R			GTAATGAGAATGCGGGGAAA
Atoh1 (P2), F	Atonal homolog 1	Sonic Hedgehog Pathway	CAACTGTGCAAGCTGAAAGG
Atoh1 (P2), R			GTACCCCGTTCACCTGTTTG
Aqp4 (P1), F	Aquaporin 4	Water transport	CCACGGTTCATGGAAATCTT
Aqp4 (P1), R			TCAGTCCGTTTGGAATCACA
Aqp4 (P2), F	Aquaporin 4	Water transport	TACACCGGTGCCAGTATGAA
Aqp4 (P2), R			TGGTCCAACCCAATATATCCA
Bdnf, F	Brain‐derived neurotrophic factor	Neurotrophin	TTGAACACGTGATCGAGGAG
Bdnf, R			TCCGCGTCCTTATTGTTTTC
Calb1 (P1), F	Calbindin	Purkinje neuron marker	GGGCAAAGAGATGATGGAAA
Calb1 (P1), R			ATCGGAATAGCAGCAGGAAA
Calb1 (P2), F	Calbindin	Purkinje neuron marker	GGAGTCAAAATGTGTGGGAAA
Calb1 (P2), R			TGTATCCATTGCCATCCTGA
Casp3 (P1), F	Caspase‐3	Proapoptotic	CTGGCAAACCCAAACTTTTC
Casp3 (P1), R			GTCCCACTGTCCGTCTCAAT
Casp3 (P2), F	Caspase‐3	Proapoptotic	AGCAGTTTTATTTGCGTGCTT
Casp3 (P2), R			CAACAGGTCCATTTGTTCCA
Cldn3, F	Claudin‐3	Tight junction marker	TTATCACAGCGCAGATCACC
Cldn3, R			ACACTTTGCACTGCATCTGG
Cldn5 (P1), F	Claudin‐5	Tight junction marker	CTGGACCACAACATCGTGAC
Cldn5 (P1), R			AGCACCGAGTCGTACACCTT
Cldn5 (P2), F	Claudin‐5	Tight junction marker	CTGGTTCGCCAACATCGT
Cldn5 (P2), R			AAGCTTCTCCTGCTCTGCTG
Dcx (P1), F	Doublecortin	Neurogenesis marker	CCTCAGGGAGTGCGTTACAT
Dcx (P1), R			ATAGCTTTCCCCTTCCTCCA
Dcx (P2), F	Doublecortin	Neurogenesis marker	TTGGTGACGACGATGTGTTT
Dcx (P2), R			TGACTCGGCATTCATTTTCA
Efnb1 (P1), F	Ephrin‐B1	Qiagen array suggestion	AAATCCGCTTCACCATCAAG
Efnb1 (P1), R			CAGGCTCCCATTGGATGTAG
Efnb1 (P2), F	Ephrin‐B1	Qiagen array suggestion	TGACCATCTTTTCCCTCCTG
Efnb1 (P2), R			GGGCAGATGATGTCCAGTTT
Erbb2 (P1), F	V‐erb‐b2 avian erythroblastic leukemia viral oncogene homolog 2	Qiagen array suggestion	CAGCACATCCACCAGGAGT
Erbb2 (P1), R			AAGGTGCCAGTGGAGACTTG
Erbb2 (P2), F	V‐erb‐b2 avian erythroblastic leukemia viral oncogene homolog 2	Qiagen array suggestion	CCCCAACACGACTCTAGCC
Erbb2 (P2), R			GGCAACGTAGCCATCAGTTT
Flk1 (P1), F	VEGF‐Receptor 2	Angiogenesis pathway	GCATCCGAAGAGCTGAAAAC
Flk1 (P1), R			ATGCCACAGACTCCTTGCTT
Flk1 (P2), F	VEGF‐Receptor 2	Angiogenesis pathway	ATCCCAGATGACAGCCAGAC
Flk1 (P2), R			ATGGCGCTAATTTGGTTCTG
Flt1 (P1), F	VEGF‐Receptor 1	Angiogenesis pathway	GAAAGGCCAAGATTTGTGGA
Flt1 (P1), R			AGTCTTTGCCGTCCTGTTGT
Flt1 (P2), F	VEGF‐Receptor 1	Angiogenesis pathway	CTACAAGCAGCCCATCACAA
Flt1 (P2), R			CGATGAATGCACTTTCTGGA
GFAP (P1), F	Glialfibrillaryacidic protein	(Bergmann) gliacell marker	ACATCGAGATCGCCACCTAC
GFAP (P1), R			GCAGATTGGAGAAGGTCTGC
GFAP (P2), F	Glialfibrillaryacidic protein	(Bergmann) gliacell marker	GCAGACCTTCTCCAATCTGC
GFAP (P2), R			CTCCACAGTCTTCACCACGA
Glut1 (P1), F	Glucose transporter 1	Energy metabolism	GTCACCATCCTGGAGCTGTT
Glut1 (P1), R			ATAGAAAACCGCGTTGATGC
Glut1 (P2), F	Glucose transporter 1	Energy metabolism	GCATCAACGCGGTTTTCTAT
Glut1 (P2), R			GTGGCATACACAGGCTGCT
Glut3 (P1), F	Glucose transporter 3	Energy metabolism	TCCCCTCAGCTGCATTCTAT
Glut3 (P1), R			CCAGAAGACAACGAGGAAGC
Glut3 (P2), F	Glucose transporter 3	Energy metabolism	GCTGGCGTGGTTAATACCAT
Glut3 (P2), R			CTCCAAGGCCTATCAGATGC
Gpr124 (P1), F	Probable G‐protein‐coupled receptor 124	Angiogenesis regulator	GCTGTGCTCATGGAACTGAG
Gpr124 (P1), R			GAGAAGAGGCAGAGCAGCAG
Gpr124 (P2), F	Probable G‐protein‐coupled receptor 124	Angiogenesis regulator	TCTGCCTCTTCTCCACCATC
Gpr124 (P2), R			CATGTGGAAGCACAGGTTCA
Hdac4 (P1), F	Histone deacetylase 4	Qiagen array suggestion	CATTGACATCCACAGCCAGT
Hdac4 (P1), R			TTCCTCGTTCTCGCACTTCT
Hdac4 (P2), F	Histone deacetylase 4	Qiagen array suggestion	TCTCTGCTTTGCTGGGAAAC
Hdac4 (P2), R			CGGAGTTGTCGTAGGGTCTC
HIF‐1a (P1), F	Hypoxia‐inducible factor 1‐alpha	Hypoxia marker	GAATGGAACGGAGCAAAAGA
HIF‐1a (P1), R			TGATTGCCCCAGGAGTCTAC
HIF‐1a (P2), F	Hypoxia‐inducible factor 1‐alpha	Hypoxia marker	TGTGTTATCTGTCGCTTTGAGTC
HIF‐1a (P2), R			TTTCGCTTTCTCTGAGCATTC
ICAM1, F	Intercellularadhesionmolecule 1	Tight junction marker	GGGGTCCATACAGGACACTG
ICAM1, R			CAGCTCGTACTTCTGCGACA
Itpr3 (P1), F	Inositol 1,4,5‐Trisphosphate Receptor, Type 3	Purkinje neuron differentiation	GTCATGGACGTGGAGTTCCT
Itpr3 (P1), R			GAGGTCAAAGAGCAGGATGC
Itpr3 (P2), F	Inositol 1,4,5‐Trisphosphate Receptor, Type 3	Purkinje neuron differentiation	TCTGCTCATGTGCATTGTCA
Itpr3 (P2), R			GGGAAGAGCGACTCATCTTTT
Lifeguard (P1), F	Lifeguard	Anti‐apoptotic	TACAACACCACATCCGTGCT
Lifeguard (P1), R			GTCGAACTTGGTCTGGAAGC
Lifeguard (P2), F	Lifeguard	Antiapoptotic	GGAGCAGGCGTGTTTACATT
Lifeguard (P2), R			TGAGGGCGCCAAAAATATAC
LOC100623510 (P1), F	Protein S100‐B‐like (no SusScrofaortholog)	Qiagen array suggestion	AGCTCATCAACAGCGAGCTT
LOC100623510 (P1), R			GCTGTCCAGTGTCTCCATGA
LOC100623510 (P2), F	Protein S100‐B‐like (no SusScrofaortholog)	Qiagen array suggestion	CAGGAGGTCGTGGACAAAGT
LOC100623510 (P2), R			GGTAACCATGGCGACAAAAG
Mbp (P1), F	Myelin Basic Protein	Myelinization marker	TGACTACAAACCGGCTCACA
Mbp (P1), R			TCCCAGCTTGAAGATTTTGG
Mbp (P2), F	Myelin Basic Protein	Myelinization marker	GGACTGTCCCTCAGCAGATT
Mbp (P2), R			GAGCCGGTTTGTAGTCAGGA
Mct1 (P1), F	Monocarboxylate transporter 1	Energy (lactate) metabolism	CCGACTTCTGGCAAAAGAAC
Mct1 (P1), R			GGCTTCTCAGCAGCGTCTAT
Mct1 (P2), F	Monocarboxylate transporter 1	Energy (lactate) metabolism	GGTGGAGGTCCTATCAGCAG
Mct1 (P2), R			GAAGGAAGCTGCAATCAAGC
Mct2 (P1), F	Monocarboxylate transporter 2	Energy (lactate) metabolism	CTCACTTGGCCTCTGTGTGA
Mct2 (P1), R			AAAGATGCCTGGCAAGAAGA
Mct2 (P2), F	Monocarboxylate transporter 2	Energy (lactate) metabolism	GGTCCCCACCCATTAGTTTT
Mct2 (P2), R			ATGGAGAGGGCTGAGGATTT
Mdk (P1), F	Midkine (neurite growth‐promoting factor 2)	Qiagen array suggestion	GAAGGCTCGGTACAATGCTC
Mdk (P1), R			TTTTCCCTTCCCTTTCTTGG
Mdk (P2), F	Midkine (neurite growth‐promoting factor 2)	Qiagen array suggestion	GGTGGCCAAAAAGAAAGACA
Mdk (P2), R			CACTCCGCAGTCCTTGCT
Neurog1 (P1), F	Neurogenin 1	Qiagen array suggestion	GCCACTCTCTGACCCCAGTA
Neurog1 (P1), R			AGGCCTGGAAAGGAGAAAAG
Neurog1 (P2), F	Neurogenin 1	Qiagen array suggestion	CTTCCCAGACGACAGCAAG
Neurog1 (P2), R			GCCAGAGCCCAGATGTAGTT
Ngf (P1), F	Nerve Growth Factor	Neurotrophin	TCAGCATTCCCTTGACACAG
Ngf (P1), R			AAGTTTGGGGTCCACAGTGA
Ngf (P2), F	Nerve Growth Factor	Neurotrophin	CAACAGGACTCACAGGAGCA
Ngf (P2), R			CTGTCGCACACCGAGAACT
Nrp1 (P1), F	Neuropilin 1 (VEGF ligand)	Qiagen array suggestion	TTCAAGAGGGGTCCTGAATG
Nrp1 (P1), R			GGCTGTTGGGGTATTTTTCA
Nrp1 (P2), F	Neuropilin 1 (VEGF ligand)	Qiagen array suggestion	TCGAAAGCTTTGACCTGGAG
Nrp1 (P2), R			CCAATATGGGGACCAACATC
Nrp2 (P1), F	Neuropilin 2	Angiogenesis pathway	GTTACTGCCTTGCGTTCCTC
Nrp2 (P1), R			CATCCTCGTAGCCCTCTCTG
Nrp2 (P2), F	Neuropilin 2	Angiogenesis pathway	CGACATGGAGTACCAGCAGA
Nrp2 (P2), R			GAGGAACGCAAGGCAGTAAC
Ntf3 (P1), F	Neurotrophin 3	Neurotrophin	AGACTCGCTCAATTCCCTGA
Ntf3 (P1), R			CTGAAGGTCCACCATCTGCT
Ntf3 (P2), F	Neurotrophin 3	Neurotrophin	CAAAACCTCCCAGACCTACG
Ntf3 (P2), R			ACAAGGCACACACACAGGAC
Ocln, F	Occludin	Tight junction marker	GACGAGCTGGAGGAAGACTG
Ocln, R			GTACTCCTGCAGGCCACTGT
p75 (P1), F	Nerve Growth Factor Receptor	Neurotrophin receptor	CGACAACCTCATCCCTGTCT
p75 (P1), R			GCTGTTCCACCTCTTGAAGG
p75 (P2), F	Nerve Growth Factor Receptor	Neurotrophin receptor	CTGCAAGCAGAACAAGCAAG
p75 (P2), R			TCTGGCTGTCCACAGAGATG
Pdgfr‐beta (P1), F	Platelet‐derived growth factor receptor beta	Tyrosinekinase receptors	CTCACCGTCATCTCCCTCAT
Pdgfr‐beta (P1), R			AGCTCACGGATTCGATCACT
Pdgfr‐beta (P2), F	Platelet‐derived growth factor receptor beta	Tyrosinekinase receptors	GAGCCATTCTCAGGCTACCA
Pdgfr‐beta (P2), R			GACATGAGGGCTTGCTTCTC
Pecam‐1 (P1), F	Platelet endothelial cell adhesion molecule	Endothelialcell marker	TTGGAAACCATGCAATGAAA
Pecam‐1 (P1), R			GGTCACTTCCACTTCCGTGT
Pecam‐1 (P2), F	Platelet endothelial cell adhesion molecule	Endothelialcell marker	ACACGGAAGTGGAAGTGACC
Pecam‐1 (P2), R			TCAGCTTTCCGGATTTCACT
Ptch, F	Patched receptor	Sonic Hedgehog Pathway	GCGTGGATGATGTTTTCCTT
Ptch, R			GCTTGAGGCATTCTCCAGTC
Pxn (P1), F	Paxilin	Angiogenesis pathway	CTCTCTCCCAGAGGGGAAAC
Pxn (P1), R			GTGGAGTGGTCTGGCTCTTC
Pxn (P2), F	Paxilin	Angiogenesis pathway	CTCCCCTGTGAACTTTCTGG
Pxn (P2), R			TTCCTGAGAAGGCAGGAGAA
Shh (P1), F	Sonic Hedgehog	Sonic hedgehog pathway	GCGACTTCCTCACCTTCTTG
Shh (P1), R			GGCTCTCTGGTCTCGATCAC
Shh (P2), F	Sonic Hedgehog	Sonic hedgehog pathway	AGCAGTTTATCCCCAACGTG
Shh (P2), R			TGTAATTGGGGGTGAGTTCC
Smo1, F	Smoothened receptor	Sonic Hedgehog Pathway	CAGCAAGATCAACGAGACCA
Smo1, R			GTGGCAGCTGAAAGTGATGA
Snf2 h (P1), F	SWI/SNF‐related matrix‐associated actin‐dependent regulator of chromatin subfamily A member 5	Purkinje and granula cell development	TACAAGGTGCCTCGAAATCC
Snf2 h (P1), R			TCATCGTTAAGGGGTTCAGC
Snf2 h (P2), F	SWI/SNF‐related matrix‐associated actin‐dependent regulator of chromatin subfamily A member 5	Purkinje and granula cell development	GAAAGGGGAGAGGCAAGAAT
Snf2 h (P2), R			TGTACCGTCCAATCTTCGTG
Syp (P1), F	Synaptophysin	Synaptogenesis marker	GGAATACCTGCAAGGAGCTG
Syp (P1), R			AGAGCACCAGGTTCAGGAAG
Syp (P2), F	Synaptophysin	Synaptogenesis marker	GTGACCTCTGGCCTCAACAC
Syp (P2), R			CTCCTTGAACACGAACCACA
TrkB (P1), F	BDNF/NT‐3 growth factors receptor	Neurotrophin receptor	TTGTGTGGCAGAAAATCTCG
TrkB (P1), R			GGTCTGAGGTTGGAGATTCG
TrkB (P2), F	BDNF/NT‐3 growth factors receptor	Neurotrophin receptor	GGGGCAATTTTGAATGAGTC
TrkB (P2), R			CGTGGTACTCCGTGTGATTG
VE‐Cad (P1), F	VascularEndothelialCadherin	Tight junction marker	AGAGCCTCATGGGGAAGAAT
VE‐Cad (P1), R			TCTGAGGAGAGGCTGAGGAG
VE‐Cad (P2), F	VascularEndothelialCadherin	Tight junction marker	AACACACCTCTGGGAAATGG
VE‐Cad (P2), R			TGTCAAAGGGTGTGCTGAAG
Vegf‐A, F	Vascular endothelial growth factor A	Angiogenesis pathway	ACATCTTCAAGCCGTCCTGT
Vegf‐A, R			ACACTCCAGACCTTCGTCGT
Vegf‐B (P1), F	Vascular endothelial growth factor B	Neurotrophin pathway	GTGAAGCCAGACAGGGTTTC
Vegf‐B (P1), R			GTGGGATGGGTGATGTCAG
Vegf‐B (P2), F	Vascular endothelial growth factor B	Neurotrophin pathway	CTCTGGCCACCAAAAGAAAG
Vegf‐B (P2), R			TCCATGGTTAGAGGCACCAC
Wnt7a (P1), F	Wingless type, member 7A	Embryogenesis and angiogenesis marker	GCCTGGACGAGTGTCAGTTT
Wnt7a (P1), R			GCTCCCCACTTTGAGCTCTT
Wnt7a (P2), F	Wingless type, member 7A	Embryogenesis and angiogenesis marker	ATCAAGAAGCCGCTGTCCTA
Wnt7a (P2), R			GGTCCTCCTCGCAGTAGTTG
Wnt7b (P1), F	Wingless type, member 7B	Embryogenesis and angiogenesis marker	CGCGAGATCAAGAAAAACG
Wnt7b (P1), R			CACTTGCACTCCAGCTTCAT
Wnt7b (P2), F	Wingless type, member 7B	Embryogenesis and angiogenesis marker	GCTACGGCATCGACTTCTCC
Wnt7b (P2), R			TCGTTGTTGTGCAGGTTCAT
ZO‐1 (P1), F	Tight junction protein 1	Tight junction marker	ATGACTCCTGACGGTTGGTC
ZO‐1 (P1), R			TGCCAGGTTTTAGGATCACC
ZO‐1 (P2), F	Tight junction protein 1	Tight junction marker	CCGCCTCCTGAGTTTGATAG
ZO‐1 (P2), R			CAGCTTTAGGCACTGTGCTG
Beta‐actin, F	Beta‐actin	Reference gene	CTACGTCGCCCTGGACTTC
Beta‐actin, R			GCAGCTCGTAGCTCTTCTCC
B2 m, F	Beta‐2‐microglobulin	Reference gene	TGAAGCACGTGACTCTCGAT
B2 m, R			CTCTGTGATGCCGGTTAGTG
Gapdh, F	Glyceraldehyde 3‐phosphate dehydrogenase	Reference gene	ACCCAGAAGACTGTGGATGG
Gapdh, R			AAGCAGGGATGATGTTCTGG
PP1a, F	Protein phosphatase 1 alpha	Reference gene	CAAGACTGAGTGGTTGGATGG
PP1a, R			TGTCCACAGTCAGCAATGGT
Rpl13A, F	60S ribosomal protein L13a	Reference gene	ATTGTGGCCAAGCAGGTACT
Rpl13A, R			AATTGCCAGAAATGTTGATGC
Tbp, F	Tatabox‐binding protein	Reference gene	ACGTTCGGTTTAGGTTGCAG
Tbp, R			CAGGAACGCTCTGGAGTTCT

List of genes, relevance and primer sequences used for Fluidigm qPCR. P1 and P2 states the two “names” given for all newly designed primer sets. Reference genes are given as the last six in the gene list.

Data were retrieved and inspected using Fluidigm's Real‐Time PCR Analysis software, version 3.0.2 and exported to GenEx5 (MultiD, Göteborg, Sweden) for data preprocessing as previously described (Skovgaard et al. 2013). Data normalization was performed to four highly stable reference genes. Using GeNorm (Vandesompele et al. 2002) and NormFinder (Andersen et al. 2004), Beta‐actin, GAPDH, RPL13A, and TBP were identified as stable expressed reference genes out of six candidates. For each primer assay, the lowest mean relative expression level was set to 1 and all data scaled accordingly, during data transformation from log2 (*C*q) to linear scale.

### Statistics

For the analysis of each of the stereological, Western blot, and Fluidigm qPCR outcomes the three postnatal sampling time points (day 0, 5, 26), the time of birth (term vs. preterm), and the diet (ENT vs. TPN) was tested using factorial analysis of variance (ANOVA) (IBM SPSS Statistics Version 22). Bonferroni post hoc tests were used to validate any significant main effects for the Western blot and stereological data, whereas specific selection criteria were applied to the qPCR data (see below). To correlate gray and white matter growth to postconceptional age (PCA) in the presentations, newborn term piglets were considered to have a PCA of 118 days and the newborn preterm pigs a PCA of 106 days (Fig. 1). The PCA of other groups were then calculated by adding the respective postnatal ages (5 or 26 days, see Fig. 1). The effect of preterm birth on volume parameters was tested by ANCOVA using the statistical software program R (version 3.0.3), where the estimated volumes for preterm piglets at postnatal days 5 and 26 were compared with the expected volumes calculated from the normal growth curves. The normal growth curves were constructed using the volume estimates from preterm postnatal day 0 (PCA = 106 days), term postnatal day 0, 5, and 26 (PCA = 118, 123, and 144 days, respectively, Fig. 1). Diet effects were tested separately for the term and preterm pigs by ANOVA. A covariance analysis was performed to test, whether the gray/white matter ratio was explained by PCA or birth type (preterm/term).

In the qPCR experiments, genes were considered to be differentially expressed if *P *<* *0.05, and if the expression changed ≥2‐fold for a single primer or ≥1.5‐fold for double primers. Due to these conservative selection criteria, no Bonferroni correction was performed on the qPCR data.

## Results

### Experiment 1

**Figure 2:**
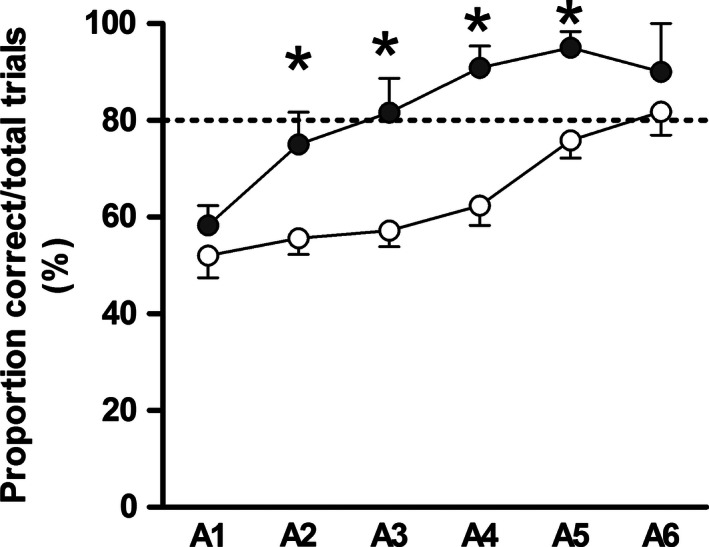
T‐maze experiment. T‐maze test using term (black symbols, *n *= 6) and preterm (white symbols, *n *= 17) pigs. Each symbol represents average performance (mean ± SEM) of all pigs and of all ten trials for each of six consecutive days (A1–A6), starting on postnatal day 15. The term pigs reached the learning criterion (80% correct choices) 3 days before the preterm pigs (*P < 0.05).

In the T‐maze, all pigs initially performed according to chance on day 15 of life [~50% correct choices (*P* = 0.09, term vs. preterm), Fig. 2]. Although all pigs improved their performance over time (*P* < 0.001), learning was significantly delayed in preterm versus term pigs that took 3 days more to reach the learning criterion (*P* < 0.01, 80% correct choices). In the T‐maze, both the latency to the choice of a reward arm (speed of decision making), and the total distance moved, decreased with time, but were similar between groups.

### Experiment 2

Organ weights, clinical chemistry, and behavioral characteristics of the pigs have been reported in a separate publication (Andersen et al. 2016). The preterm pigs showed a long series of signs that indicate their immature organ development at birth, including reduced body growth and liver and gut weights together with mild hypothermia, hypoxia, and hypoglycemia during the first few days after birth (Andersen et al. 2016).

#### Cerebellar volumes

**Figure 3:**
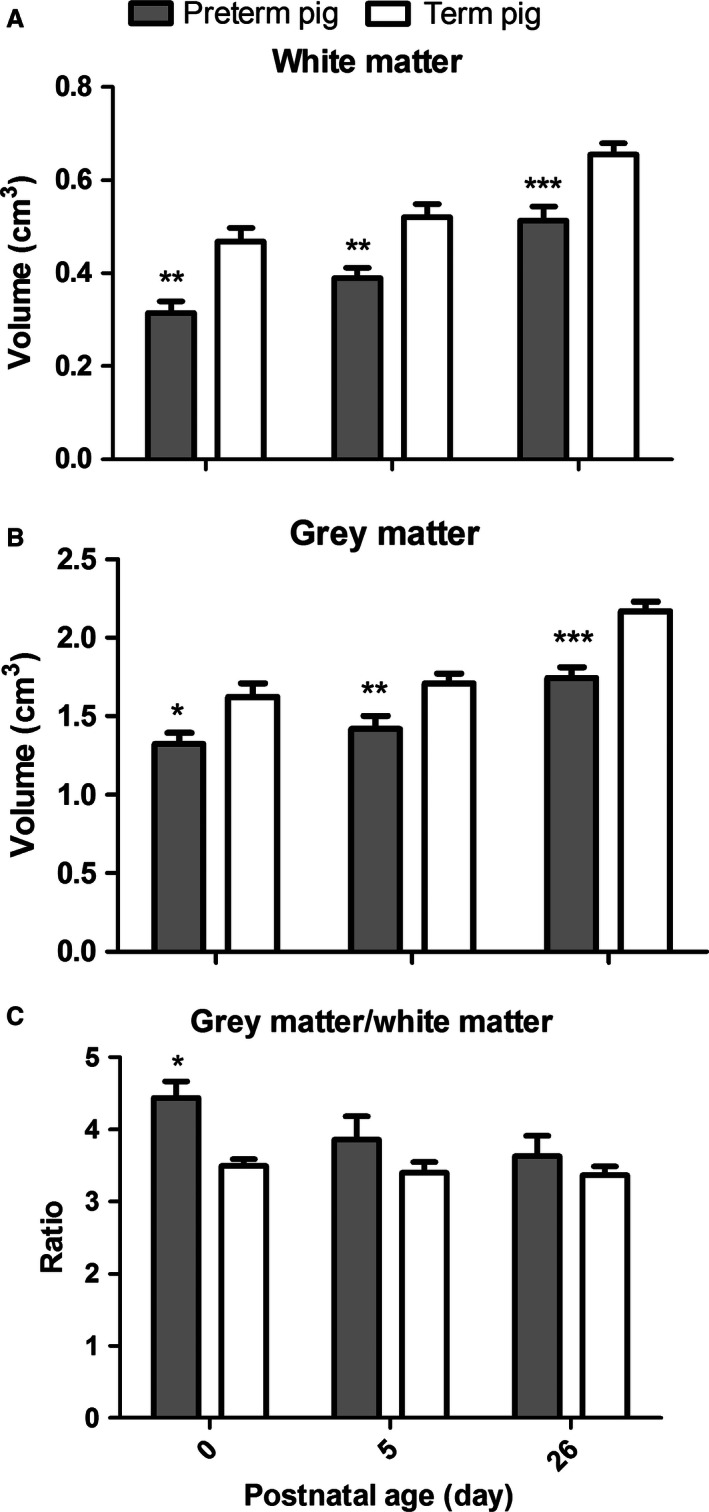
Postnatal comparison of cerebellar volumes. For both white matter (A) and gray matter (B), the cerebellar volumes were smaller for preterm (gray bars), relative to term (white bars) pigs at all measured ages (*P < 0.05, **P < 0.01, ***P < 0.001). The gray/white matter ratio (C) was higher in preterm pigs at birth (*P < 0.05).

Macroscopical evaluation of the brains collected from both preterm and term pigs at all three ages (0, 5, and 26 days) and after different treatments for the first 5 days (TPN, ENT) revealed no visible signs of brain injury, for example, white matter injury, hemorrhage, or apparent inflammatory lesions. There were no significant effects of the diet interventions (TPN, ENT) during the first 5 days on any of the investigated volumetric parameters (data not shown). Because of the minimal effects of diet during the first 5 days, data for the two diet regimens were pooled in the subsequent statistical analyses, as shown in Figure 3.

**Figure 4:**
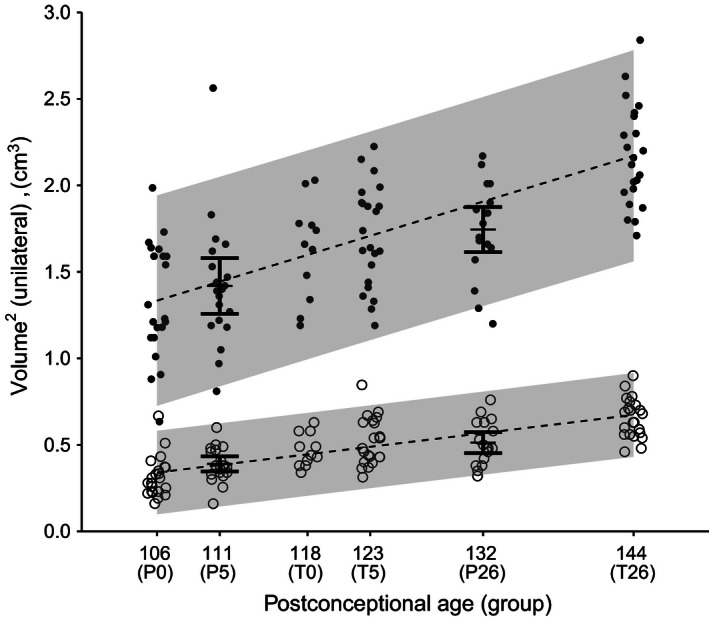
Development of cerebellar gray and white matter volumes. Each symbol shows a volume estimate for a single pig (black circles = gray matter, open circles = white matter. Dotted lines display the linear fit of volume as a function of postconceptional age of PCA 106, 118, 123, and 144 corresponding to preterm postnatal age 0, and term postnatal ages 0, 5, and 26 days. The shaded gray area shows the prediction intervals for the linear fits. The black bars shows means ± confidence intervals for PCA 111 and 132, corresponding to preterm postnatal age 5, and 26 days. The observed means for PCA 111 and 132 were not significantly different from the expected values calculated from the growth curves (*P *> 0.05). The total relative growth from 90% gestation to 26 days in term pigs was 108 and 64% for white and gray matter, respectively.

Comparison between preterm and term pigs showed significant differences for white matter (Fig. 3A) and gray matter (Fig. 3B) at postnatal day 0, 5, and 26, respectively (*P* < 0.001–0.05). The gray to white matter ratio was significantly higher in preterm pigs, relative to term pigs on day 0 (*P* < 0.05, Fig. 3C). Across preterm and term pigs, the cerebellar gray and white matter volumes both increased significantly with age (*P* < 0.0001). We therefore generated a linear fit through P0, T0, T5, and T26 representing the normal growth of gray and white matter, respectively, and tested whether the observed means for P5 and P26 differed from the expected means. The observed means for P5 and P26 were not significantly different from the expected (*P* > 0.05). Analyzed across preterm and term piglets according to their PCA, the estimated gray matter volume increased by 0.02 cm^3^ per day, corresponding to an increase of 65% from 12 days before normal term to 26 days after birth (1.33 ± 0.32 to 2.17 ± 0.29 cm^3^, Fig. 4). The estimated white matter volume increased by only 0.01 cm^3^, yet due to the smaller absolute levels of white matter, this corresponded to a total growth of 108% from 12 days before term to 26 days after birth (0.31 ± 0.11 to 0.65 ± 0.11 cm^3^). The gray/white matter ratio decreased with increasing PCA (*P* < 0.01, *R* ^2^ = 0.09, data not shown). This decrease was not different between terms and preterms (*P* = 0.09), but rather correlated to PCA (*P* < 0.05, data not shown).

#### Sonic hedgehog pathway proteins

Neither Shh protein level nor its downstream pathway factors, Smo and Gli‐1, showed any significant differences between preterm and term pigs at any of the three time points (data not shown). We found no effect of the diet interventions during the first 5 days (TPN, ENT) on the sonic hedgehog pathway, nor on the postnatal development during the first 26 postnatal days (data not shown).

#### qPCR gene expression analyses

Ten of the 84 tested Qiagen array genes fulfilled our predefined selection criteria at day 26. Eight genes were significantly upregulated in preterm versus term pigs (*P* < 0.05), here presented with their corresponding fold changes (FC): Ephrin‐B1 (+1.5), Neuropilin 1 (+1.5), Sonic Hedgehog (+1.6), Histone Deacetylase 4 (+1.6), V‐erb‐b2 avian erythroblastic leukemia viral oncogene homolog 2 (+1.6), Vascular endothelial growth factor A (+1.7), Adenosine receptor 2a (+2.2), and Midkine (+2.2). Two genes were significantly downregulated (*P* < 0.05): Neurogenin 1 (0.6) and Protein S100‐B‐like (0.4). These ten target genes were subsequently included in the qPCR analyses of day 0 (*n* = 21) and day 26 (*n* = 56) animals.

The Fluidigm qPCR comprised a total of 49 genes, with six reference genes and 33 additional genes of relevance for cerebellar development. The qPCR results showed no consistent significant interactions among age (0 vs. 26 days), diet (TPN vs. ENT), or birth type effects (term vs. preterm) for any of the tested genes. Consequently, the results could be summarized as the gene expression ratios for day 26 versus day 0, and for preterm versus term pigs on day 0 and day 26, respectively (Table 2). The majority of analyzed genes showed either no differential regulation or regulation below our inclusion criteria (*P* < 0.05 and concomitant gene expression fold change >2‐ or >1.5‐fold or <0.5‐ or <0.75‐fold for single or double primer sets, respectively). Genes showing increased expression between birth and day 26 were Bdnf, Ntf3, and Hif‐1a (*P* < 0.01), whereas significant decreases were observed for p75, Atoh‐1, Icam‐1, Dcx, Efnb1, and Nrp1 (*P* < 0.01). Preterm pigs showed upregulation of five genes at birth (Mdk, Ntf3, p75, Efnb1, and Dcx, +40–90%, *P* < 0.01), relative to term pigs, and three of these continued to show upregulation at day 26 (Mdk, p75, Ntf3, +40–70%, *P* < 0.01). No genes were downregulated in preterm pigs, relative to term pigs, neither at birth nor after 26 days.

**Table 2:** qPCR results

Gene category/annotation	Gene	Day 26/Day 0 (Preterm + Term)	Preterm/term (Day 0)	Preterm/term (Day 26)
Neurotrophic factors/receptors	Bdnf	2.1***		
	Ntf3 (P1)	1.5*	1.5***	
	Ntf3 (P2)	2.1***	1.9***	1.7**
	p75 (P1)	0.7***	1.5***	1.4**
	p75 (P2)	0.7***	1.5***	1.7***
Neurogenesis/angiogenesis	Dcx (P1)	0.4***	1.4***	
	Dcx (P2)	0.4***	1.5***	
	Efnb1 (P1)	0.7***	1.4***	
	Efnb1 (P2)	0.6***	1.4***	
	Mdk (P1)		1.6***	1.7***
	Mdk (P2)		1.4**	1.6***
	Nrp1 (P1)	0.7**		
	Nrp1 (P2)	0.7*		
Hypoxia	Hif‐1a (P1)	1.4*		
	Hif‐1a (P2)	1.5***		
Sonic hedgehog pathway	Atoh1	0.5***		
Tight junction	Icam1	0.4***		

Ratios of mean values of Day26/Day0 and Preterm/Term for differentially expressed genes by Fluidigm qPCR. *P < 0.05, **P < 0.01, ***P < 0.001. Blank cell = not significant (*P* > 0.05) or 0.75/0.5 < mean expression value ratio <1.5/2 for 2 or 1 primer sets, respectively. Note that for p75, Efnb1, Dcx, and Mdk, a borderline mean value ratio of 1.4 was included.

## Discussion

We recently demonstrated that preterm pigs show distinct behavioral and motor coordination delays relative to term pigs during the first weeks after birth (Cao et al. 2015; Andersen et al. 2016). Together with the inferior performance of preterm pigs in the T‐maze in this study, this verifies the functional neurodevelopmental delay in preterm pigs. It was surprising that these functional differences were not associated with more clear structural or molecular changes in the preterm pig cerebellum. Except for a few potentially important differences, preterm and term pigs did not differ markedly in cerebellum morphology, white to gray matter ratios, and the protein abundance and expression of a large number of genes considered important for brain maturation. Our endpoints were investigated in a state of relatively slow growth for both preterm and term pigs. This was caused by a need to standardize the feeding regimens between preterm and term piglets for optimal comparison, thus term piglets were fed at the same relatively low feed intake, as for preterm pigs, leading to relatively slow growth. In addition, the cesarean section, lack of initial sow rearing (colostrum uptake) and artificial rearing may have contributed to slow growth, not only for the preterm but also for the term piglets (Cao et al. 2015; Andersen et al. 2016).

The lack of clear effects of preterm birth and the first enteral nutrition on our endpoints indicates that the preterm pig brain is relatively mature and resilient at 90% gestation, despite that other critical organs (lungs, gastrointestinal, liver) are clearly immature at this time (Sangild et al. 2013; Caminita et al. 2015; Andersen et al. 2016). The preterm 90% gestation piglet is considered to have an overall survival capacity that is similar to 28–30 week‐old infants (Sangild et al. 2013), but specifically for the developing brain, the 90% gestation piglet may be more similar to “late preterm infants” (e.g., 34–37 weeks gestation). Regardless, it remains difficult to compare structural and functional organ development in relation to birth among different species because the age‐related maturation varies among organs and also among different regions and cell populations within the same organ.

One of the questions addressed in the present study was whether postconceptional (PCA) effects or postnatal effects (environmental triggers) appeared to be most important as regulators of postnatal brain (i.e., cerebellar) development in the preterm pig. This distinction is important for the interpretation of the relatively few, significant structural and molecular differences that we observed in our study. In preterm pigs, we observed reduced gray and white matter cerebellar volumes during the first postnatal month, and higher gray to white matter ratio at birth. Correspondingly, there was a relatively larger increase in white matter than in gray matter volumes from 12 days before normal term to 26 days after term birth, when data were viewed relative to the date of conception. Postconceptional age, rather than being born preterm or term, seemed to explain the major part of the variation of the gray/white matter ratio. In contrast to the brain, maturation of the gut is always affected immediately after birth, preterm or term, partly mediated by the exposure to nutritional and microbiota triggers (Sangild et al. 2013). These environmental triggers have short‐ or long‐term effects on the developing gut depending on each specific structure or function (Hansen et al. 2016). Perhaps the brain is better protected by the meninges and the blood–brain barrier, and postnatal triggers are therefore less likely to have an immediate and strong effect on CNS development, beyond the genetic developmental program mainly determined by the PCA. Comparison of brains from groups of 12 d‐old preterm pigs with those of term newborn pigs would have helped these evaluations but such PCA‐matched groups were not included in our study. In the postnatal period, we chose to collect brains on day 5 for both groups because here preterm pigs become fully mobile, have a stable metabolism and respiration and show marked gut maturational responses to enteral feeding (Sangild et al. 2013; Cao et al. 2015; Andersen et al. 2016; Hansen et al. 2016).

The fact that cerebellar white matter grows faster than gray matter in the perinatal period of preterm pigs suggests that processes involving myelination or glial cell neurogenesis is important in the preterm cerebellum during the first postnatal month. These results are consistent with the observed increased cerebral white matter myelination in normal pigs (Winter et al. 2011) and with the abnormal cerebellar white matter development in preterm infants (Hart et al. 2010). Several recent MRI‐based studies of preterm infants have demonstrated significant correlations between cerebellar volume and cognitive performance in infancy, early childhood, adolescence, or early adulthood (van Kooij et al. 2012; Nosarti et al. 2014; Keunen et al. 2016). Furthermore, sex differences in cognitive performance (boys performing worse) of 30‐month‐old preterm infants also showed significant correlations with cerebellar volume estimates (Skiold et al. 2014), emphasizing the importance of this brain region, not only for motor purposes, but also for higher ranking brain functions.

The majority of the vast number of carefully selected neurodevelopmental genes did not differ in mRNA expression levels between preterm and term pigs. Nevertheless, mRNA levels of Neurotrophin 3, p75 neurotrophin receptor, and Midkine were consistently higher in preterm pigs throughout the first 4 weeks, whereas Doublecortin and Ephrin‐B1 were higher at day of birth. For these genes, cerebellar expression therefore seems to decrease significantly over the extra 12 days of intrauterine life for the term piglets. Our comprehensive list of analyzed genes was based on a combination of thorough literature searches and a commercial neurogenesis array screen but we cannot exclude that potentially interesting genes could have been missed. We recently repeated the cerebellum qPCR experiment using the same gene list for a new set of 3 week‐old term and preterm pigs and results were very similar to this study with a relatively small number of genes differentially regulated between preterm and term pigs (A. Bergström, K. Ryom, K. Skovgaard, T. Thymann & P.T. Sangild unpublished results).

All ten genes that differed in expression between birth and 26 days showed no difference in response to gestational age at birth (preterm, term) or introduction of enteral nutrition (ENT) during the first 5 days, relative to pigs fed total parenteral nutrition (TPN). It was recently demonstrated that 10 days of TPN in preterm pigs leads to decreased cerebellar volume, reduced motor activity and decreased myelination, relative to full enteral milk feeding (120–200 mL/kg/day) (Choudhri et al. 2014). Consequently, 5 days of minimal enteral nutrition with bovine colostrum (0–60 mL/kg/day) may have been insufficient to affect cerebellar development, relative to TPN, in this study.

The observed qPCR fold changes expressed relative to postconceptional age suggests that preterm birth and its postnatal consequences stimulate a regulatory cascade of events that involves accelerated white matter growth, probably to catch up with inadequate neuronal signaling. This process of axonal myelination would be mediated by specific angiogenesis and neurogenesis markers (Mdk, Efnb1, Dcx) and neurotrophic factors (Ntf3, p75) (Kadomatsu et al. 2014) and is relevant in the context of preterm infants (Brew et al. 2014). Axon myelination by oligodendrocytes in the developing white matter is a very energy demanding process and has been shown to correlate strongly with angiogenesis and tissue oxygenation (Yuen et al. 2014). This may involve increased myelinization of central cerebellar neurons, for example, Purkinje and granula cells, supporting the maturation of motor, balance, and coordination functions (Wyatt et al. 2005).

Sonic hedgehog (Shh), expressed by the Purkinje cells during development, is believed to play a crucial role in the differentiation of both Purkinje neurons and Bergman glia cells (Rakic and Sidman 1970; Dahmane and Ruiz i Altaba 1999), thereby playing a central role in cerebellar development (Volpe 2009b). Shh and Bdnf are known to have a mitogenic effect on the granule cell precursors of the so‐called external granular layer (Haldipur et al. 2011, 2012). In preterm infants, advancing postnatal age negatively affects cerebellar Shh pathway activity (Haldipur et al. 2011). This could not be demonstrated in our study on Shh proteins in pigs during the final part of gestation or in the postnatal period of preterm pigs.

The apparent absence of macroscopic white matter injury in both groups, and lacking differences in proteins related to the sonic hedgehog pathway, or in gene expressions related to hypoxia, ischemia, tight junction integrity, glucose/lactate metabolism, apoptosis, or myelination suggest that the preterm pig cerebellum is relatively resilient to the physiological stressors just after preterm birth and to environmental stimuli such as enteral feeding and bacterial colonization. The delay in acquisition of basic motor skills, reduced physical activity, and inferior balance, coordination and cognitive capacities in preterm pigs (Cao et al. 2015; Andersen et al. 2016) may therefore be determined mainly by an age‐related developmental delay, rather than by inappropriate responses to environmental factors such as enteral feeding, gut bacterial colonization, inflammation, dysmetabolism, or hypoxia after preterm birth. This is important because it indicates the degree to which optimal care and associated morbidities can be expected to influence brain development in preterm neonates, beyond the maturation occurring as a result of advancing age pre‐ and postnatally. Future model studies in preterm pigs should investigate more brain regions and environmental factors with analyses matched for both PCA and chronological age for longer periods.

The 90% gestation preterm pig may have several advantages as a model to study brain development in late preterm infants, relative to models in sheep or rodents. Both term (Elmore et al. 2012; Liu et al. 2014; Radlowski et al. 2014) and preterm pigs (Andersen et al. 2016) show great cognitive potential and the pig may be the only model animal that displays a perinatal synaptogenetic timing that is similar to that in infants (Dobbing and Sands 1979). Pigs have large litter sizes and better allow for long‐term medical and nutritional interventions, relative to preterm lambs or rodents. An ability to combine shortened gestational age at birth with the postnatal consequences of preterm birth (e.g., respiratory, metabolic, gut, and immunological challenges) is critical for a good model of preterm birth.

## Conflicts of Interest

R.M. van Elburg is employed at Nutricia Research
