# Erratum

**DOI:** 10.14814/phy2.12954

**Published:** 2016-08-24

In 1, the surname of author Per T. Sangild was misspelled as “Sanglid.”

The online version of the article has been corrected.

## References

[1] Bergstorm, A. , S. S. Kaalund , K. Skovgaard , A. D. Andersen , B. Pakkenberg , A. Rosenorn , R. M. van Elburg , T. Thymann , G. O. Greisen , and P. T. Sangild . 2016 Limited effects of preterm birth and the first enteral nutrition on cerebellum morphology and gene expression in piglets. Physiol Rep 4:e12871. 27462071 10.14814/phy2.12871 PMC4962075

